# Enzymatic and transcriptomic analysis reveals the essential role of carbohydrate metabolism in freesia (*Freesia hybrida*) corm formation

**DOI:** 10.7717/peerj.11078

**Published:** 2021-03-19

**Authors:** Li Ma, Suqin Ding, Xueqing Fu, Zi Yan, Dongqin Tang

**Affiliations:** 1 Department of Landscape Architecture, Shanghai Jiao Tong University, Shanghai, China; 2 Plant Biotechnology Research Center, Fudan-SJTU-Nottingham Plant Biotechnology R&D Center, School of Agriculture and Biology, Shanghai Jiao Tong University, Shanghai, China

**Keywords:** Freesia, Corm formation and development, Starch and sucrose metabolism, Corm swelling, Metabolizing enzymes, Transcriptome

## Abstract

Starch and sucrose metabolism plays a crucial role in the formation and development of bulbs in bulbous plants. However, these mechanisms remain unclear and unexplored in the corms of *Freesia hybrida*. Herein, we investigated the dynamics of the major form of carbohydrates and related enzyme activities and profiled the transcriptome of freesia corms at four developmental stages with the aim to reveal the relation between the expression of genes involved in the metabolism of carbohydrates and the accumulation of carbohydrates in corm developmental stages for further exploring the mechanism on the starch and sucrose metabolism regulating the formation and development of corms in *F. hybrida*. The content of starch, sucrose and soluble sugars followed an overall upward trend across the corm developmental stages. Activities of the adenosine diphosphoglucose pyrophosphorylase, starch branching enzyme and β-amylase generally followed the pattern of the starch and sucrose levels. Activities of sucrose phosphate synthase increased from corm formation till the initial swelling stage and subsequently reached a plateau. Activities of invertase and sucrose synthase peaked at the later rapid swelling stage. These suggested that the starch and sucrose dynamics paralleled corm swelling under the action of metabolic enzymes. A total of 100,999 unigenes were assembled in the transcriptomic analysis, and 44,405 unigenes of them were annotated. Analysis based on Clusters of Orthologous Groups suggested that carbohydrate transport and metabolism (9.34% of the sequences) was prominent across the corm developmental process. In total 3,427 differentially expressed genes (DEGs) were identified and the enrichment analysis detected starch and sucrose metabolism as a critical pathway in corm development, especially at the rapid swelling stage. Further, DEGs encoding key carbohydrate-metabolizing enzymes were identified and correlated to enzyme activities and carbohydrate accumulation. The results construct a valuable resource pool for further molecular-level studies, which are helpful for metabolic regulation of carbohydrates and improvement in *F. hybrida*.

## Introduction

Freesia (*Freesia spp.*), a species of Iridaceae family and indigenous to South Africa (Wang, 2006), is one of the most popular cut flowers in the world for its pleasant flower aroma and diverse flower colors. Freesia is a bulbous herbaceous perennial and it sprouts in the autumn and blooms in winter to spring. It was introduced from the native habitat into other countries and subject to hybridization breeding, producing assorted commercialized modern freesia (*F. hybrida*). Corms are the propagative organ, thus the quality of corms determines the healthiness of the plant and then the ornamental value of freesia. Previous studies suggest that development of storage roots was paralleled with complex physiological activities including carbohydrate, enzyme, and hormone dynamics (Miao et al., 2016a; Miao et al., 2016b; Vishnevetsky, Zamski & Ziv, 2000). A wealth of studies regarding this have been conducted on bulbous flowers such as lilies (Jing, Liu & Zhou, 2010; Li et al., 2014; Xia, Zheng & Huang, 2004) and tulips (Miao et al., 2016a; Miao et al., 2016b; Ohyama et al., 1988), whereas freesia remains unexplored according to our knowledge.

Starch is a major storage form of carbohydrate and an energy carrier in plant storage organs. For instance, starch content was proposed to be a key trait for judging lily bulb quality (Wu et al., 2012). During lily bulblet formation, starch in mother bulb degraded in favor of daughter bulb development (Li et al., 2014); thereafter, a continuous accumulation of starch was observed till bulblet maturation. Apart from being a labile source of carbon and energy (Martínez, Vieitez & Corredoira, 2015), sucrose is the major form of carbohydrate for long distance translocation and serves as a substrate for starch production in many plants, as well as a signaling (Rook et al., 1998) and osmotic regulating substance (Gao et al., 2018b). Sucrose was reported to be the major form of soluble sugar accounting for potato tuberization (Fernie & Willmitzer, 2001) and cell division and enlargement during tulip stolon development (Miao et al., 2016a). A concomitant accumulation of starch and sucrose was observed across tube and bulblet development (Fernie & Willmitzer, 2001; Xia, Zheng & Huang, 2004). In *Gladiolus hybridus* bulbs, starch and sucrose serve as not only storage but also signaling substances regulating the source–sink relationship of the bulbs (Qian, He & Yi, 2007; Yuan et al., 2008). At the initial morphogenesis stage of the new bulb, the metabolism of starch and sucrose was at a low level. As shoots developed, sucrose was accumulated in shoots and transported to the bulbs where it converted to starch, leading to rapid swelling of the bulb. As shoots withered, sucrose accumulation in each organ slightly declined and the accumulation of starch in the storage organ reach a plateau (Yuan et al., 2008). Therefore, the metabolic level of starch/sucrose was closely correlated to the lifecycle of *Gladiolus hybridus*. In tulip bulbs, starch breakdown into soluble sugars arising from elevated *α*-amylase activity in response to coldness (Lambrechts, Rook & Kolloffel, 1994), which could enhance the cold tolerance of the storage organs (Lee et al., 2012; Thalmann & Santelia, 2017). As a result, a rapid elongation of flower stalk as well as a faster growth of shoot and daughter bulbs were observed (Lambrechts, Rook & Kolloffel, 1994). Nevertheless, biological functions of starch and sucrose remains to be explored in freesia. In some corm/bulb producing species in Iridaceae, fructans are the major reserve form of carbohydrate (Almeida et al., 2015; Hendry, 1993). In bulbs of *Lachenalia minima* (Hyacinthaceae) and tulips, fructans co-occur with starch to fulfill different functions (Kamenetsky et al., 2003; Orthen, 2001a). By contrast, freesia corms may not produce fructose as indicated by Orthen (2001b) that a freesia species (*Freesia viridis*) did not store fructans in the corm.

Starch and sucrose metabolism is closely related to a series of key enzymes. Sucrose synthase (SuSy) catalyzes the reversible synthesis of sucrose, but hydrolytic activity is suggested to play a leading role (Miao et al., 2016a). Sucrose phosphate synthases (SPS) promote sucrose synthesis and invertases (INV), including neutral invertases (NI) and acid invertases (AI), are responsible for irreversible degradation of sucrose. SuSy and INV synergistically break down sucrose into fructose and glucose, which can subsequently join in starch synthesis pathways (Li et al., 2014; Zhang et al., 2016). Adenosine diphosphoglucose pyrophosphorylase (APGase) initiates the synthesis of starch by catalyzing the formation of adenosine diphosphate glucose (ADPG), a precursor of starch synthesis (Rouhier & Usuda, 2001). Subsequently, soluble starch synthase and granule-bound starch synthase catalyze the starch synthesis using the substrate, ADPG. Starch branching enzymes (SBE) add branches to glucan to form amylopectin. Amylases (AMY) is a key enzyme that cleaves starch into soluble sugars to sustain plant growth. Miao et al. (2016a); Miao et al. (2016b)) revealed that the development of *Tulipa edulis* stolons was accompanied with a sugar consumption and starch production via the action of enzymes. Vishnevetsky, Zamski & Ziv (2000) found that content of carbohydrates was strongly correlated to corresponding enzyme activities in *Nerine sarniensis* bulbs at different developmental stages. The significance of metabolic enzymes in carbohydrate metabolism in developmental bulbs was further revealed in oriental hybrid lily ‘Sorbonne’ (Wu et al., 2013) and *Gladiolus hybridus* (Yuan et al., 2008).

Still, there is a lack of information in molecular mechanisms related to carbohydrate metabolism in freesia corm development. Genes related to enzymes involved in starch and sucrose metabolism remain unexplored. In view of this, in the current study, the dynamic changes of levels of starch and sucrose and key enzymes involved were monitored and the transcriptome was profiled during freesia corm development to reveal functions of major carbohydrates regulating freesia corm development. The next-generation high-throughput sequencing method was employed in this study to sequence the transcriptome of freesia corms. This sequencing method applies not only to plants with known genomes but also to those lack of reference genomic sequences, and has already been successfully attempted in diverse plants for transcriptomic profiling (Onda & Mochida, 2016).

To our knowledge, this is the first thorough investigation of starch and sucrose metabolism across corm development in freesia at the physiological and transcriptomic level, which is of special importance for quality control of corms and informative for development of proper cultivation and breeding practices of freesia.

## Materials & Methods

### Plant materials

*F. hybrida* ‘SN Huangjin’ was planted in late October, 2016 in a farm on Minhang campus, Shanghai Jiao Tong University, China. Corm tissues were first sampled at the 60th day after mother corm embedment and the following samples were collected at an interval of 10 days, resulting in 14 sampling events (i.e., 60, 70, 80, 90, 100, 110, 120, 130, 140, 150, 160, 170, 180 and 190 d after planting). Our previous work detailed the biological characteristics of the plant and divided the developmental process of the new corm into four stages, i.e., the formation (60–90 d), initial swelling (90–120 d), rapid swelling (120–140 d) and maturation (140–190 d) stages (Ding et al., 2019). Pictures of corms at different developmental stages were displayed in Table S1. The plants were thoroughly rinsed with water to remove dirt and the newly developed corms were collected for analysis. At each sampling event, 10 healthy corms of similar size from 10 individual plants were ground and divided into multiple portions prior to flash freezing with liquid nitrogen for storage (−80 °C), waiting for analysis.

### Determination of carbohydrate content and related enzyme activities

Contents of the carbohydrates and activities of corresponding enzymes were determined in corms collected at 14 sampling events. Sucrose was determined according to Deng et al. (2006). Soluble sugars and starch were detected based on anthrone method and colorimetric assay of iodine, respectively (Miao et al., 2016a). Enzymes were extracted as described by Keller & Ludlow (1993). Among them SPS and SuSy activities were evaluated using procedures by Wei et al. (2007) and Datir & Joshi (2016), AI and NI activities by Crusciol et al. (2017) and SBE activities by Fu et al. (2009). Beta-AMY activities were determined by spectrophotometry. Three biological replicates for each parameter were performed.

### RNA extraction, cDNA library construction, sequencing and assembly

Corms sampled at 60, 90, 120 and 190 d, representing four developmental stages of new corms (e.g., formation, initial swelling, rapid swelling and maturation stages) respectively, were selected for transcriptomic analysis. Three biological replicates for each stage were used. RNA was extracted using RNApre Pure Plant Kit (Tiangen Biotech, Beijing, China) following the manufacture’s protocol. An aliquot of 80 µL DNase I composed of 10 µL DNase and 70 µL RDD was pipetted into the spin columns, which stood still for 15 min at the room temperature, to remove the genomic DNA. An equal portion of RNA extracted from each developmental stages (sampled at 60, 90, 120 and 190 d) was subject to library construction, sequencing and assembly. Quality of RNA was detected using a NanoDrop 2000 spectrophotometer (Thermo Scientific, Waltham, MA, USA) and gel electrophoresis (1%). Clear bands and appropriate A260/A280 (1.9–2.1) and A260/A230 (>2.0) values obtained in this study suggested that the RNA quality was good enough for library construction. RNA-seq was performed by BioMarker Biotechnology Corporation (Beijing, China) based on Illumina (CA, USA). Raw data were filtered to generate clean reads which were de novo assembled by Trinity to obtain unigenes (Grabherr et al., 2011).

### Gene functional annotation

Unigenes were aligned, by BLASTx (*E*-value ≤ 1e−5), to NCBI Non-redundant Protein (Nr), Gene Ontology (GO), Clusters of Orthologous Groups of proteins (COG), Kyoto Encyclopedia of Genes and Genomes (KEGG), euKaryotic Clusters of Orthologous Groups (KOG), Swissprot and evolutionary genealogy of genes: Non-supervised Orthologous Groups (eggnog) databases for functional annotation. Unigenes were searched against the Pfam database by HMMER with an *E*-value ≤ 1e−10. Functional annotation by GO terms was carried out by Blast2Go (Gotz et al., 2008). The KEGG pathway annotation was performed online using KEGG Automatic Annotation Server (https://www.genome.jp/tools/kaas/).

### Analysis of differentially expressed genes (DEGs)

The transcript abundance of each unigene was calculated and normalized to fragments per kilobase of transcript per million mapped reads (FPKM) value, which is the most commonly used method to estimate gene expression levels (Trapnell et al., 2010). DEGs between different developmental stages were screened using DESeq software with raw count matrices as input (Anders & Huber, 2010). Obtained *P* values was corrected using Benjamini–Hochbergs method to account for multiple tests using the false discovery rate (FDR). Genes with FDR <0.001 and absolute expression fold change (log2 scaled) ≥4 were deemed to be significantly differentially expressed. Identified DEGs were then subject to GO database for functional analysis and to KEGG for identification of metabolic pathways.

### Quantitative real-time polymerase chain reaction (qRT-PCR) analysis of DEGs of key enzymes involved in sucrose and starch metabolism

DEGs of key enzymes involved in sucrose and starch metabolism were retrieved from KEGG results and their expression was verified by qRT-PCR. RNA was extracted and purified as described above. The first-strand cDNA was synthesized using Prime Script™ RT reagent kit with gDNA Eraser (TaKaRa, Dalian, China) and then used as the template for qRT-PCR. Briefly, the RNA was reversely transcribed in the 10 µL of reaction media containing 0.5 µg of total RNA applying random hexamer primers to synthesize the first-strand cDNA. Primers, designed using Primer 5.0 software, were provide by Qingxi Biology (Shanghai, China). Real time qRT-PCR was conducted in a reaction volume of 20 µL containing 10 µl of SYBR, 7.4 µL of double-distilled H_2_O, 0.8 µL of each primer and 1µL of cDNA mix. Relative gene expression levels were acquired using the 2 − ΔCT method after normalizing to actin (the internal reference gene) (Kilambi et al., 2013). Three biological replicates and three technical replicates within each biological sample were included for qRT-PCR.

## Results

### Starch and sucrose dynamics

### Starch metabolism and corresponding enzyme dynamics

**Figure 1:**
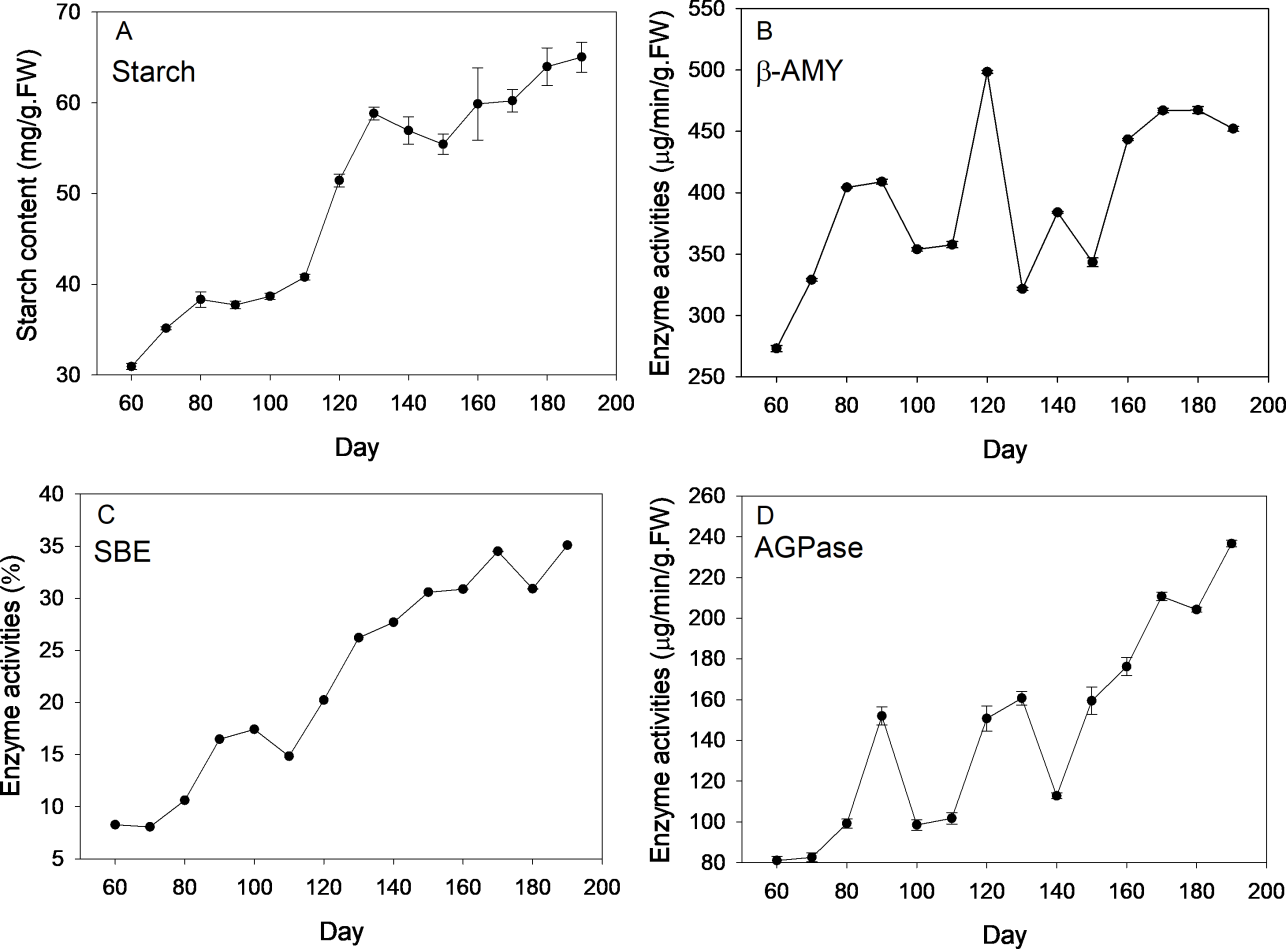
Changes of starch content and activities of corresponding enzymes during freesia corm development. (A) Starch, (B) *β*-AMY, (C) SBE, (D) AGPase. AMY, amylase; SBE, starch branching enzyme; AGPase, adenosine diphosphoglucose pyrophosphorylase; FW, fresh weight.

Description of four stages of corm development was displayed in Table S1. Dynamics of starch content and the enzyme activities are shown in Fig. 1. Over the new corm developmental stages, starch content saw an overall increasing trend with the minimum (30.94 mg/g FW) and maximum value (65.04 mg/g FW) appearing, respectively, at the beginning and the end of the sampling event. Starch content increased slowly within 110 days since mother corm embedding. Thereafter, the content rose rapidly reaching ∼60 mg/g FW from 110 to 130 d (transition from initial swelling stage to rapid swelling stage), followed by a slightly decrease downward (∼130–150 d) and subsequent increase at the maturation stage (∼150–190 d).

Beta-AMY activities were rather dynamic. Specifically, *β*-AMY activities increased from 273 to 408 µg/min/g FW during the corm formation stage (60–90 d). After transient decrease, the enzyme activity increased sharply and peaked at 120 d (appearance of flower bud), reaching 498.33 µg/min/g FW, which was followed by marked decrease in the next sampling event and ensuing increase with fluctuations during maturation stage. Throughout the corm lifecycle, an overall increasing trend of SBE activities was observed, especially at the rapid swelling stage. During the maturation stage (150–180 d), the increasing rate of enzyme activity was almost flat. The value of the highest enzymatic activity was ∼4 fold that of the minimum at the beginning of sampling. Overall, APGase activities increased across the corm development stages, especially during the formation (60–90 d) and maturation stages (140–190 d), while fluctuations were observed during corm swelling (110–140 d). The minimum (∼80 µg/min/g FW) and maximum (∼240 µg/min/g FW) values appeared at the first and last sampling event, respectively.

### Sucrose metabolism and corresponding enzyme dynamics

**Figure 2:**
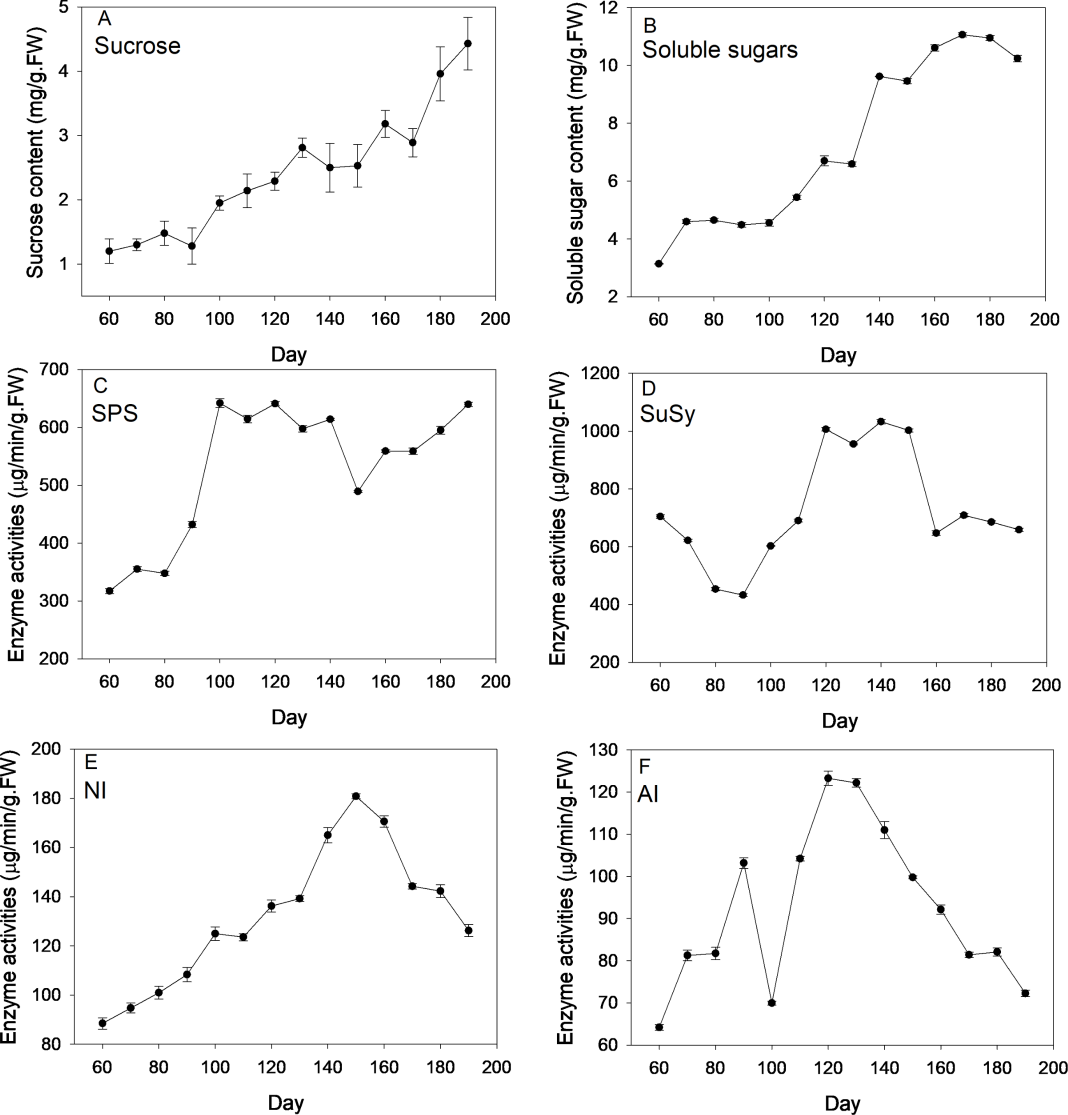
Changes of sugar content and activities of corresponding enzymes during freesia corm development. (A) Sucrose, (B) Soluble sugars, (C) SPS, (D) SuSy, (E) NI, (F) AI. SPS, sucrose phosphate synthase; SuSy, sucrose synthase; NI, neutral invertase; AI, acid invertase; FW, fresh weight.

Figure 2 depicts changes of sugar content and corresponding enzyme activities across new corm development. On average, levels of sucrose and soluble sugars followed an increasing trend across the corm developmental process. By comparison, the increasing trend was more pronounced for sucrose than for soluble sugars for which more fluctuations were observed. At the formation stage (60–90 d), sucrose and soluble sugars both kept at a relatively constant level and then increased rapidly at times during corm swelling. Even at the maturation stage, sucrose content rose rapidly to reach a maximum (4.43 mg/g FW), which was 3.69 times of the lowest value (1.2 mg/g FW) at the first sampling event. For soluble sugars, the maximum and minimum values were 3.14 and 11.06 mg/g FW, respectively.

SPS activities kept relatively constant (∼350 µg/min/g FW) during the formation stage (60–90 d), then increased sharply reaching maximum (∼640 µg/min/g FW) during corm swelling (100–140 d) and subsequently kept relatively stable except a marked drop at 150 d. Comparatively, the profile of SuSy activities exhibited a slightly different pattern. The enzymatic activity showed monotonous decline from 705 to 433 µg/min/g FW during the formation stage, then rose rapidly arriving at maximum (∼1006 µg/min/g FW) during the swelling period and finally dropped back to the beginning level when corms began mature. NI activities increased continuously from 60 d (88.47 µg/min/g FW) to 150 d (∼180 µg/min/g FW) followed by a sustaining drop arriving at 126.19 µg/min/g FW at the end. Averagely, AI activities followed an initial increasing and subsequent decreasing trend as corms grew except the presence of a drop at 100 d. The maximum and minimum activities were 64.2 and 123.27 µg/min/g FW, respectively.

### Correlations between starch and sucrose content, enzyme activities and corm size

Table 1 showes that activities of SPS, AGPase and SBE were positively and significantly correlated with sucrose and corm diameter (*P* < 0.01), which all were significantly correlated to starch content. Besides, NI exhibited a positive and significant relationship with starch content and corm dimeter (*P* < 0.01). Also, the correlation between SBE and NI and AGPase, and AGPase and *β*-AMY were significant (*P* < 0.01). By contrast, no significant association between NI, AI and *β*-AMY was observed. AI and SuSy showed no significant correlation with any other parameter at all.

**Table 1:** Pearson correlation between starch and sucrose content, enzyme activities and corm size.

	NI	AI	*β*-AMY	SPS	SuSy	AGPase	SBE	Sucrose content	Starch content	Diameter
NI	1									
AI	0.428	1								
*β*-AMY	0.327	0.148	1							
SPS	0.591^*^	0.372	0.478	1						
SuSy	0.622^*^	0.601^*^	−0.047	0.439	1					
AGPase	0.486	0.045	0.689^**^	0.497	0.100	1				
SBE	0.800^**^	0.154	0.548^*^	0.651^*^	0.392	0.878^**^	1	.		
Sucrose content	0.573^*^	0.000	0.559^*^	0.682^**^	0.256	0.856^**^	0.879^**^	1		
Starch content	0.760^**^	0.255	0.585^*^	0.662^**^	0.458	0.857^**^	0.957^**^	0.921^**^	1	
Diameter	0.839^**^	0.269	0.524	0.677^**^	0.522	0.815^**^	0.971^**^	0.889^**^	0.980^**^	1

**Notes.**

* Significance level of 0.05. ** Significance level of 0.01.

NI neutral invertase AI acid invertase AMY amylase SBE starch branching enzyme AGPase adenosine diphosphoglucose pyrophosphorylase SPS sucrose phosphate synthase SuSy sucrose synthase

### Transcriptomic profiling and unigene annotation and functional classification

The sequencing statistics based on a mixed cDNA library of freesia corms at different developmental stages are summarized in Tables S2 and S3. After data filtering, 82.54 Gb of clean reads were generated with Q30 ≥ 89.73 and GC ∼48% averagely. Totally 100,999 unigenes (Acce. No GIYA00000000) were generated after assembly with a mean length of 772 nt. The N50 was 1,507 nt and 21,476 unigenes were longer than 1,000 nt. On average, 63% of the genes were uniquely mapped.

**Figure 3:**
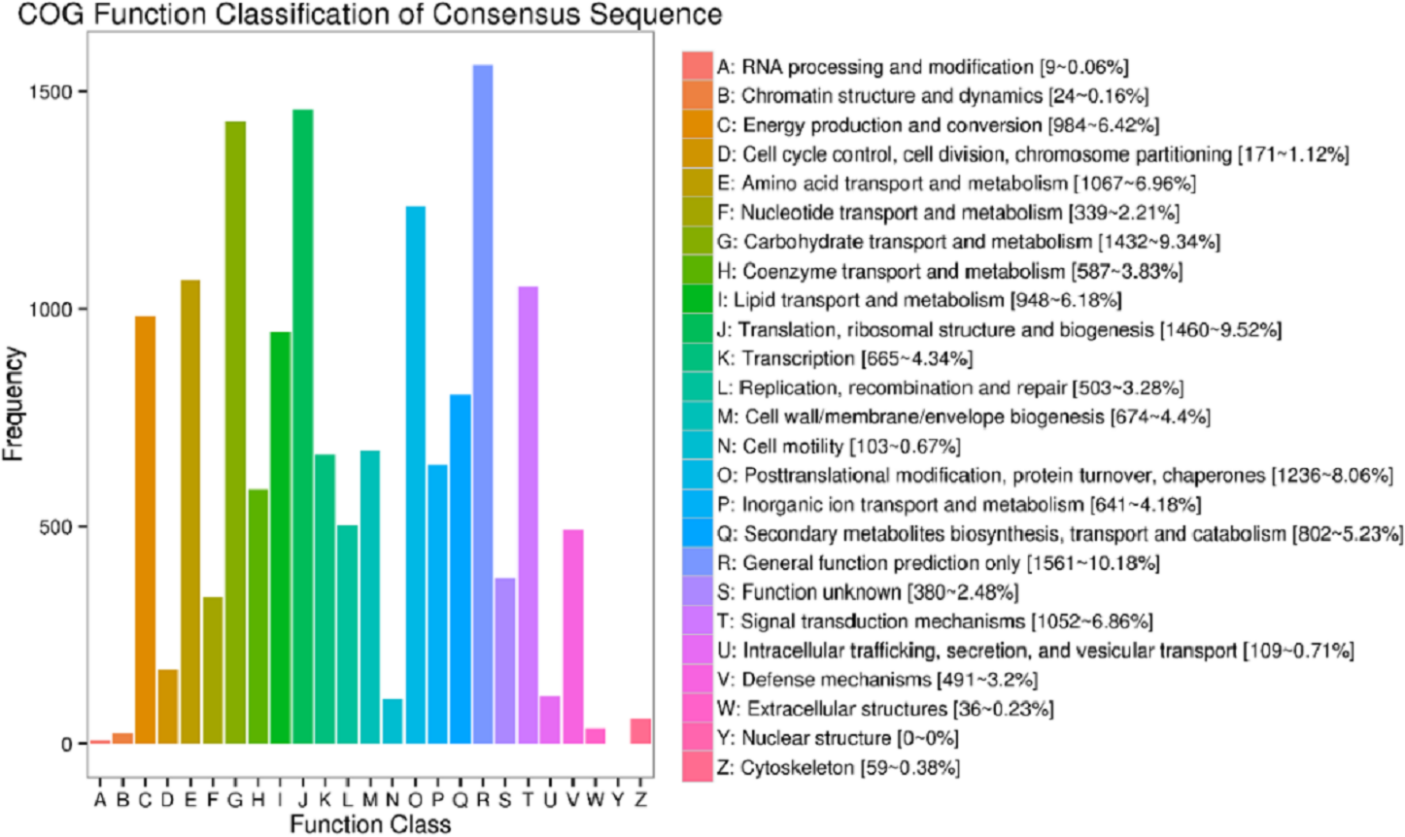
COG function classifications of unigenes in freesia corms from the mixed library of four developmental stages.

In the current study, totally 44,405 unigenes were annotated with 41,175 annotated in Nr, 13,753 in COG, 25,142 in GO, 15,743 in KEGG, 23,015 in KOG, 24,441 in Swissport and 28,454 in Pfam (Table S4). Figure 3 shows that 13,753 sequences were classified into 24 COG categories, covering majority of life activities. Apart from “general function prediction only” (1,561 unigenes, 10.18% of annotated) and “translation, ribosomal structure and biogenesis” (1,460 unigenes, 9.52%), “carbohydrate transport and metabolism” term took the central position with 1432 unigenes annotated (9.34%).

**Figure 4:**
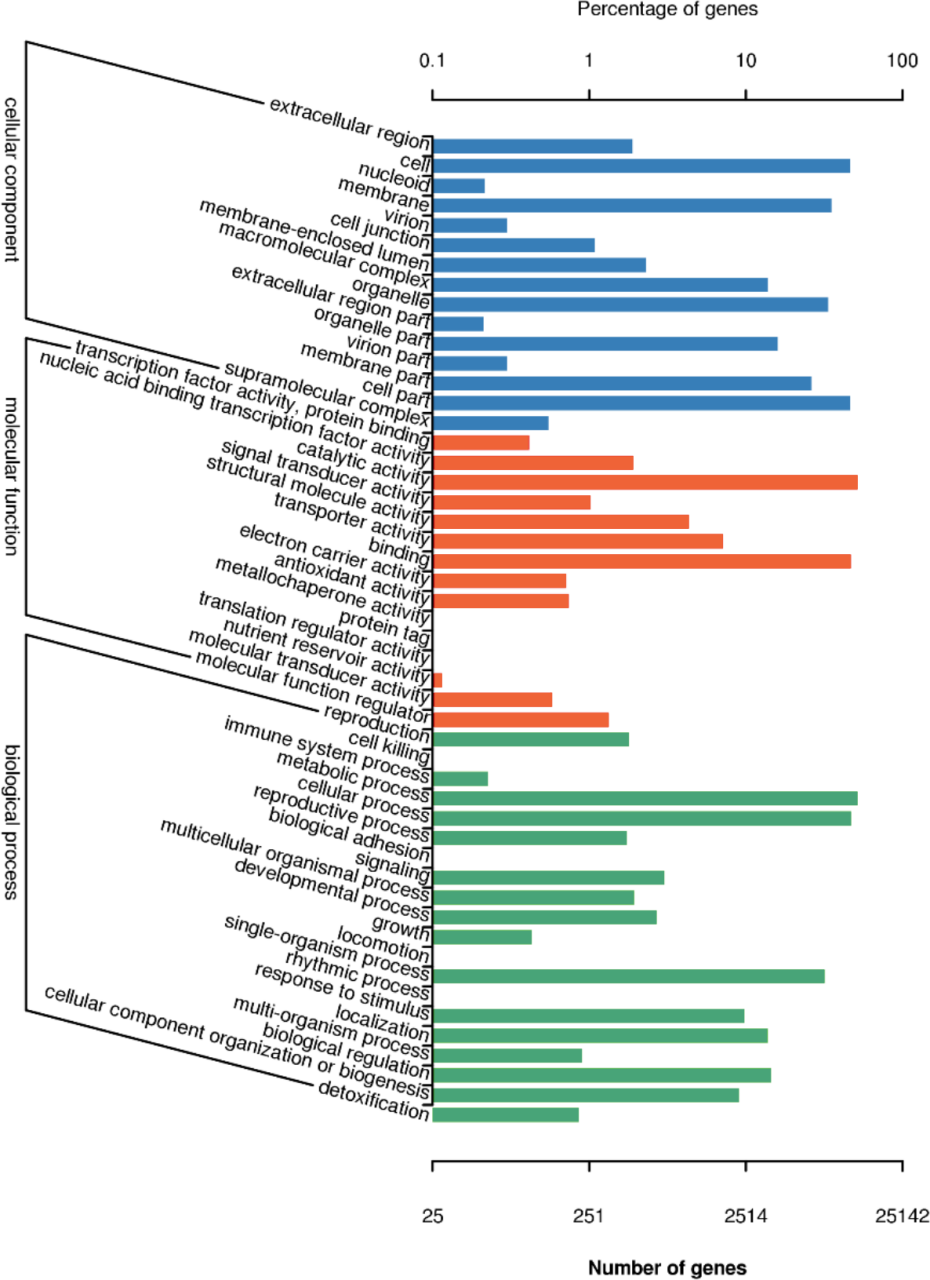
GO function classification of of unigenes in freesia corms from the integrated library of four developmental stages. The *y*-axis represents the subgroups in the three GO annotation, while the *x*-axis represents the percentage of genes matching the specific category within each of the primary categories.

There are 25,142 unigenes annotated in GO database, which were classifies into three big functional categories, i.e., cellular component, molecular function and biological process (Fig. 4). The subcategories in the principle category of cellular component was most represented by “cell”, “membrane”, “cell part” and “organelles”. Within the principle category of molecular function, most genes participated in activities related to catalysis and binding. In the principle category of biological process, genes were mostly involved in metabolic process, cellular process and single-organism process, indicative of occurrence of important metabolic and cellular activities in freesia corms. Notably, a considerable portion of genes were assigned to reproductive processes and development processes. All of these annotation information provides insights into investigation of potential genes involved in developmental processes of freesia corms.

### Distribution and function assignment of DEGs

**Figure 5:**
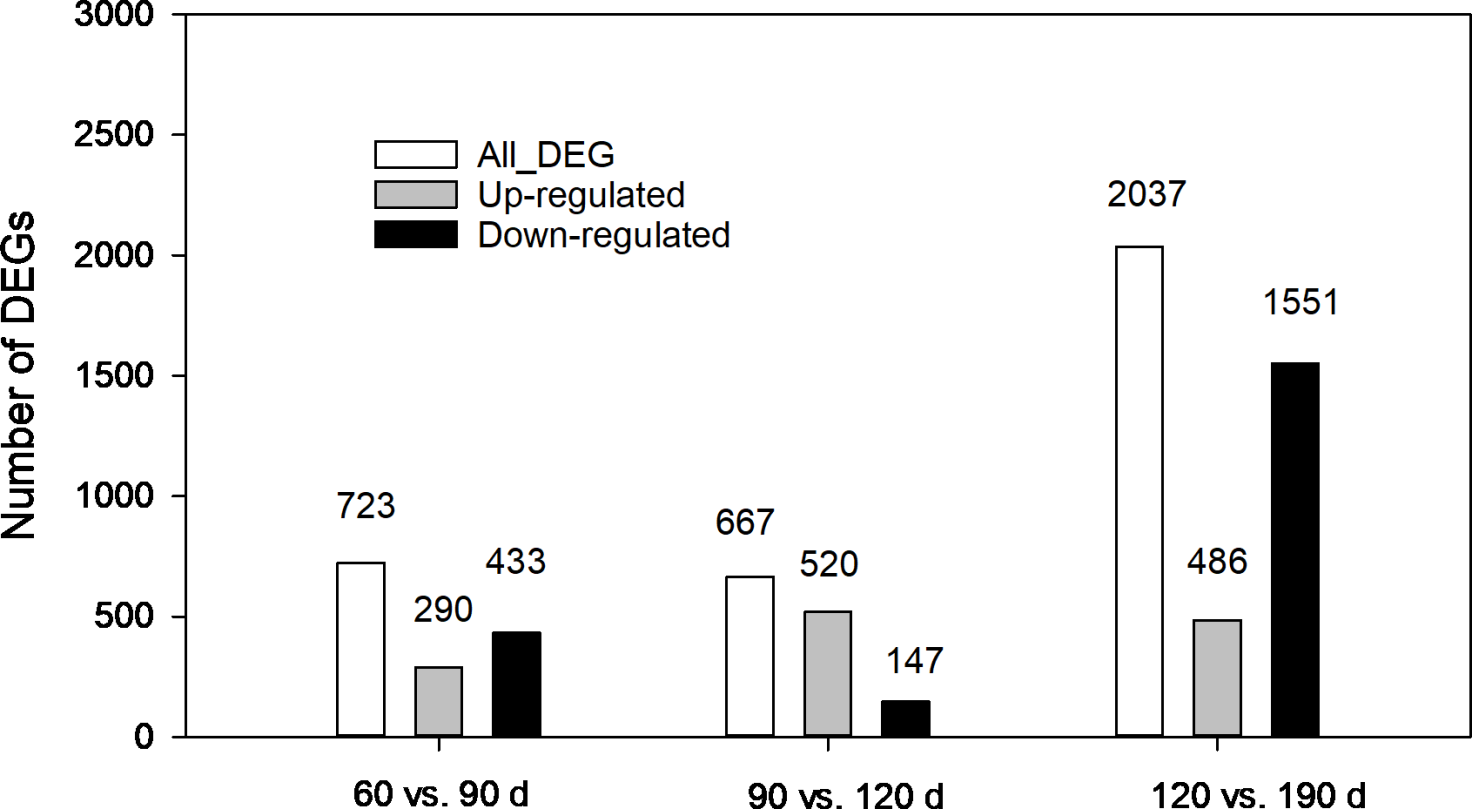
Statistics of differentially expressed genes (DEGs) at different development stages of corms.

The expression levels of unigenes at different developmental stages were compared. As shown in Fig. 5, 3,427 DEGs in total from three pairwise comparisons were detected with the largest number distributed in the third pair (120 vs.190 d), where 2,037 DEGs were identified with 1,551 down regulated and 486 upregulated. In comparison, 723 (290 up-regulated and 433 down-regulated) and 667 DEGs (520 up-regulated and 147-down regulated) were found in other two pairs (60 vs. 90 d and 90 vs. 120 d, respectively). The contrasting results suggested that more complex biochemical activities may occur during the late developmental stages especially during the rapid swelling period.

GO and COG functional and KEGG enrichment pathway analysis was performed to gain further insights into DEGs. The major categories of GO terms in three pairwise comparisons (Fig. S1) were similar to that for the combined unigenes at four developmental stages as described in Fig. 4. Notably, DEGs between 120 and 190 d were enriched in “nutrient reservoir activity” pathway, which was void in other two comparisons. Comparatively, differences in COG enrichment profiles of DEGs in three comparisons were more pronounced (Fig. S2). Within the 60 vs. 90 d comparison, the term “posttranslational modification, protein turnover, chaperones” was at the top, followed by “carbohydrate transport and metabolism” and “general function prediction only”. In the 90 vs. 120 d comparison, the top representative terms were “signal transduction mechanisms”, “carbohydrate transport and metabolism” and “posttranslational modification, protein turnover, chaperones” sequentially. While within the 120 vs. 190 d comparison, “carbohydrate transport and metabolism” ranked the first, further suggesting that carbohydrate metabolic pathway might be most active during late stages of corm development. Pathway enrichment analysis revealed top 20 enriched pathways of DEGs in Fig. S3. Generally, DEGs were, though in small quantity (<5 or 10), significantly enriched in terms like riboflavin metabolism and diterpenoid biosynthesis in 60 vs. 90 d comparison, linoleic acid metabolism and monoterpenoid biosynthesis in 90 vs. 120 d comparison, and anthocyanin biosynthesis in 120 vs. 190 d comparison. Notably, DEGs enriched in starch and sucrose metabolism in the 120 vs. 190 d comparison was quantitatively superior to other pathways, attenuating the greater activity of starch and sucrose metabolism in late developmental stages, in line with the results from GO and COG analysis.

### Expression profiles of major DEGs involved in carbohydrate metabolism

Combining data from the literature and a keyword search in RNA-seq annotation generated a total of 39 DEGs encoding six carbohydrate-metabolizing enzymes investigated in this study, i.e., SPS, AGPase, SBE, *β*-AMY, SuSy and INV. The expression of these genes was displayed in Table S5. These DEGs genes were generally differentially expressed across the corm developmental stages.

**Figure 6:**
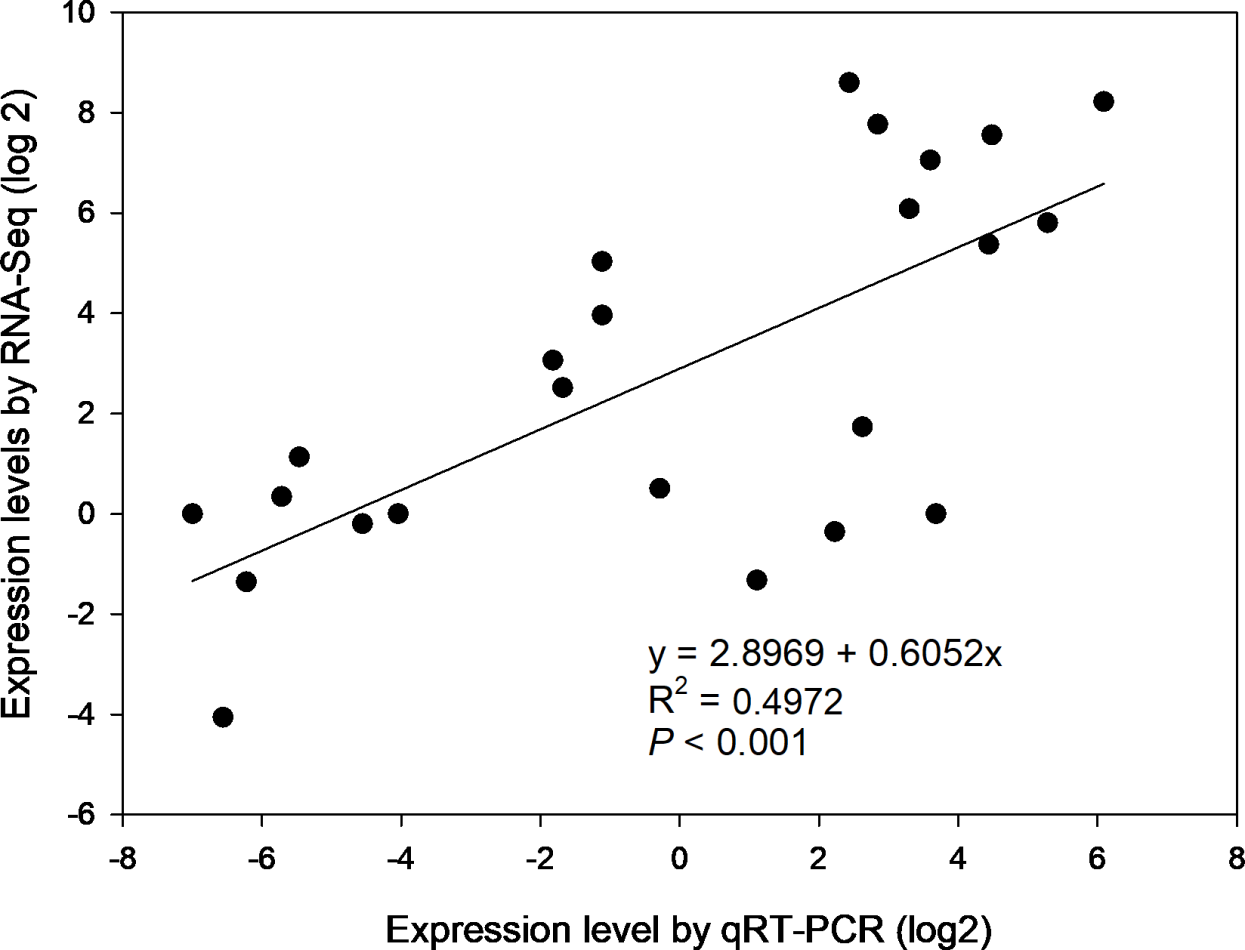
The correlation of relative gene expression determined from RNA-seq and qRT-PCR data.

To confirm the gene expression profiles of the enzymes from RNA-seq results, 6 of 39 DEGs were selected for qRT-PCR, i.e., *β*-AMY1 (c62599.graph_c1), INV2 (c87855.graph_c0), SPS1 (c91035.graph_c1), SuSy (c93394.graph_c2), SBE4 (c78179.graph_c0) and AGPGase5 (c93923.graph_c0). Primer sequences of the genes for PCR were specified in Table S6. The results of agarose gel electrophoresis evidenced that all 6 primer pairs amplified a single band (Fig. S4). Overall, the expression profiles of selected genes revealed by qRT-PCR matched that by RNA-seq across four developmental stages (Fig. S5). Correlation analysis shows that the gene expression levels from qRT-PCR and RNA-seq were positively and significantly related (*r*^2^ = 0.4972, *p* < 0.001) (Fig. 6), suggesting that the FPKM values were accurate for estimation of expression of the DEGs.

To identify key DEGs involved in carbohydrate metabolism in freesia corms, we correlated all 39 DEGs with carbohydrate-metabolizing enzyme activities and carbohydrate content; accordingly, actively expressed homologues, such as *β*-AMY2, INV (1,2 and 4), SuSy, SBE4, SPS and APGase (2 and 5), which showed expression patterns resembling those of corresponding enzyme activities and carbohydrate accumulation were determined to be critical genes involved in starch-sucrose metabolism in freesia corm swelling.

## Discussion

### Dynamic changes of carbohydrates in the developing corm via the action of enzymes

Our previous work showed that the new corm formed ∼60 day after mother corm embedment. Subsequently, corms expanded with a concomitant weight and size gain (Ding et al., 2019). Different from *tulipa* (Miao et al., 2016a) and lilies (Li et al., 2014), with daughter bulbs appearing at the base of the parent bulbs, freesia showed an initial formation of a main corm at the joint of the parent corm and stem and later multiple tiny corms at the base or top of the main corm (Table S1). Therefore, at harvest, a main corm with several smaller corms was obtained.

Similar to *Lilium davidii* var. *unicolor* (Li et al., 2014) and *Lycoris sprengeri* (Chang et al., 2013), freesia exhibited an overall increasing sucrose and starch content in developing corms. Sucrose pool in the corm was replenished by photoassimilates, which in turn serve as the substrate for corm starch replenishment. At the formation stage, levels of sucrose, total soluble sugars and starch were relatively at a low level owing to the great nutritional need from mother corms for leaf development, and great photosynthate need for aerial biomass, allowing less soluble sugars allocated to new corms. After ∼90 days of planting, sucrose and starch accumulated simultaneously at the initially swelled corms. Sucrose played a crucial role in potato tuber formation (Fernie & Willmitzer, 2001). It was postulated to be a critical factor for bulblet morphogenesis in lilies (Li et al., 2014), and was also reported to be responsible for induction and swelling of *Lilium sargentiae* bulblet (Gao et al., 2018b). The considerable level of sucrose observed in the current study thereby may be a major trigger of new corm emergency and swelling. Till this time point, a smaller portion of sugars converted to starch in corms of freesia. When the corm continued swelling, sucrose was unremittingly replenished by mother corms and photosynthetic apparatuses and the resultant increase of sucrose and soluble sugars in the corm laid a material basis for cell morphogenesis in the corm. Meanwhile, the new corm continued to act as a metabolic sink for energy input. Near the rapid swelling stage (∼120 d), starch content accumulated sharply, likely because more photosynthates or sucrose were transformed to starch in corms for storage after cell morphogenesis. At the end of rapid swelling stage (∼140 d), freesia proceeded to full flowering phase when tiny corms emerged at the base of the main corm (see Table S1); meanwhile, the main corm acted as a temporary source for tiny corm growth, the accumulation of starch hence slowed down. Since corms become mature, total soluble sugar content tended to be stable and starch content continuously increased towards the end (190 d), similar to *Narcissus* scales (Ruamrungsri et al., 1999) and lily stolons (Miao et al., 2016a). With energy reserved, the harvested mature corm will act as source to support the formation of the next-generation corm. Taken together, like lilies (Sun et al., 2011; Sun, Li & Li, 2005; Wu et al., 2013), starch and sucrose metabolism also plays a center role in formation and development of freesia corms.

Sucrose levels were closely related to corresponding enzymes (Table 1). In the initial and rapid swelling period, although SPS activities surged, SuSy activities rose faster together with the enhancement of INV activities, which catalyzed the hydrolysis of sucrose and then compromise the sucrose synthesis by SPS, leading to a slow increase of sucrose content. At the corm maturation stage, SPS showed high activities while INV and SuSy activities declined, leading to sucrose uprise. SPS activities were paralleled with that of sucrose before 100 d but afterwards it reached a plateau, in contrast to the steady increase of sucrose content. One possible explanation is that a high level of sucrose may inhibit the increase of SPS activities according to Koch (1996). Correlation analysis indicated an insignificant correlation between SuSy and sucrose (Table 1), in contrast to the inverse correlation observed in tulip stolons (Miao et al., 2016a), suggesting that decomposition effect of SuSy on sucrose was not pronounced herein. In the current study, activities of INV, especially AI, increased remarkably when corms entered the rapid swelling stage, in line with the finding that INV was very active in rapidly elongated meristems and rapid developed young organs or tissues where a concomitant slow accumulation of sucrose was observed (Arai, Mori & Imaseki, 1991). The subsequent decline of INV activities during corm maturation coincides with the statement in previous studies that INV activities tended to decline as related organs aged (Zhang & Li, 2002).

Starch metabolism is mainly regulated by APGase, SBE and *β*-AMY, and the relationship between them was significant in freesia corms (Table 1). APGase, a rate-limiting enzyme, catalyzes the formation of a precursor (ADPG) required for starch synthesis (Rouhier & Usuda, 2001). Profiles of APGase activities, though more dynamic, generally paralleled with that of the starch. SBE is an important enzyme for amylopectin formation. Both starch content and SBE dramatically increased as the corm developed, revealing that that amylopectin may be an important type of starch in the freesia corm, which needs to be further investigated. Herein, *β*-AMY activities increased initially together with starch, while it declined during bulb maturation when the nutrient requirement of freesia shoot diminished and starch mainly served as a storage form of hydrocarbon, suggesting that *β*-AMY may play an important role regulating and poising forms of hydrocarbons in freesia corms, consistent with the results observed on lily bulbs (Sun, Li & Li, 2005). Overall, dynamic changes of related enzymes implied that starch production exceeded consumption. Sucrose cleavage also contributes to the substrate for starch production (Bánfalvi et al., 1996). Consequently, starch levels presented an increasing trend across the new corm development and it was the dominant form of carbohydrates in the present study.

### Transcriptomic profiling disclosed essential roles of starch and sucrose metabolism during freesia corm swelling

Up to date, freesia genome sequencing is lacking. Although it is known that flowers of *F. hybrida* ‘Pink Passion’ and *F. hybrida* ‘Jintong’ yielded a total of 74,192 and 74,660 unigenes, among which 42,934 and 53,906 were annotated, respectively (Huang et al., 2018; Tang et al., 2018), transcriptomic sequences vary in different plant organs, and there is no enough information to illustrate the molecular mechanism of the corm development in freesia. To our knowledge, this is the first report on freesia corms using RNA-seq. In the present work, RNA-seq libraries for four developmental stages of freesia corms yielded 100,999 unigenes, larger than that in the previous studies. Of them, 44,405 unigenes (44%) were annotated while about 56% were not, resulting from the limitation of genomic sequence information in freesia. On average, in the integrated library, 9.3% of annotated COG were connected to carbohydrate transport and metabolism (Fig. 3). Meanwhile, GO analysis of unigenes directed “metabolic process” and “developmental process” important terms with gene enrichment, consistent with the involvement of genes related to carbohydrate transport and metabolism in freesia corm development. Additionally, GO and COG functional classification of DEGs pinpointed the enrichment of DEGs between the rapid swelling (120 d) and maturation stages (190 d) in “carbohydrate transport and metabolism” or “nutrient reservoir activity” categories. Further, starch and sucrose metabolism was a significantly enriched KEGG term in the 120 vs. 190 d comparison but not in other two pairs. The 120 vs. 190 pair accounted for the largest portion of DEGs, and 76% were down-regulated, while 78% of DEGs in the 90 vs. 120 d comparison were upregulated. Taken together, starch and sucrose metabolism was an essential metabolic pathway across the whole developmental process and was particularly active at the rapid swelling stage in freesia corms. As corms matured, activities of carbohydrate metabolism declined concurrent with wilting of the aboveground plant, more energy was then stored in corms acting as the next “pool”.

Transcriptome analysis of carbohydrate metabolism during bulb development, though relatively less addressed, was carried out in some bulbous or tuberous plants. Zhang et al. (2016) performed transcriptome analysis of sucrose metabolism in onion (*Allium cepa* L.) bulbs and found 7% of COG annotated unigenes were involved in carbohydrate transport and metabolism. And KEGG analysis of DEGs revealed that “starch and sucrose metabolism” was the primary metabolism pathway and was most active at rapidly expanding stage. Similarly, Li et al. (2014) conducted transcriptome analysis in *Lilium davidii* var. *unicolor* bulblets and reported that 8% of annotated COG was associated to carbohydrate transport and metabolism. KEGG analysis of DEGs indicated that starch and sucrose metabolism was the principle pathway and was most active during bulblet formation stage. Additionally, the close association of carbohydrate metabolism with storage root development was also revealed on *Lycoris sprengeri* (Chang et al., 2013), sweet potato (*Ipomoea batatas*). (Zhang et al., 2017), and *Sagittaria sagittifolia* (Gao et al., 2018a). In the present work, our findings were generally in line with the above results, confirming the important role of starch and sucrose metabolism in corm swelling via transcriptomic profiling method.

### Critical genes involved in starch and sucrose metabolism during corm development

Correlation analysis shows that the gene expression levels from qRT-PCR and RNA-seq were positively and significantly related, suggesting that the FPKM values were accurate for estimation of DEGs expression. We thus compared of FPKM profiles to that of carbohydrate accumulation and/or enzyme activities and assigned *β*-AMY2, INV (1,2 and 4), SuSy, SBE4, SPS, APGase (2 and 5) critical genes in starch and sucrose metabolism. The relative expression level of SBE4 was low before corm maturation but then increased by dozens of folds at 190 d, consistent with trend of starch accumulation, seemingly indicating that SBE4 could be essential for starch accumulation during corm maturation. The high expression level of AGPase (2 & 5) at 120 d and 190 d and SBE4 at 190 d, respectively, suggest that their coordinated expression may regulate starch accumulation in the storage organ. INV converts sucrose to glucose and fructose, which join the starch synthesis pathway. INV (1, 2 & 4) genes were dramatically upregulated from formation to rapid swelling stages followed by a rapid downregulation, congruent with profiles of associated enzymes, indicating the important role of INV in corm development. Only two *β*-AMY encoding genes were derived from the transcriptome data. The expression profile of *β*-AMY2 generally followed that of the enzyme activities. The low expression of *β*-AMY2 favors starch accumulation. Although not strictly consistent, overall trend in the expression profiles of SPS (1-10) and that of the enzyme activities and sucrose accumulation generally matched, indicating that the continuous sucrose accumulation lead to corm swelling and provided energy for reproductive growth. Only one SuSy was annotated here and its FPKM profile was opposite to that of the enzyme activities which was in line with the qRT-PCR result, suggesting possible sequencing errors or a possible converse regulation since SuSy is a bi-directional enzyme.

Considerable homologues unigenes (AGPase 2, AGPase 5 and AGPase 6 INV1, INV 2 and INV 4, etc.) peaked at 120 d (the rapid swelling stage), suggesting that the starch and sucrose metabolism was the most active in this stage. By comparison, it was found that the gene expression of some other homologues listed in Table S5 did not follow that of corresponding enzyme activities or carbohydrate variations, suggesting that, functions of the homologues may not be consistent. Also, gene expression occurs generally prior to enzyme synthesis and carbohydrate accumulation, thus, expression of these homologues may not play a decisive role in the transcriptional regulatory network. Similar inconsistences between gene expression levels and activities of starch metabolic enzymes were also observed in *Lycoris sprengeri* bulbs (Chang et al., 2013) and *Tulipa edulis* stolons (Miao et al., 2016a). Here we screened some critical genes involved in starch and sucrose metabolism in freesia and their roles remain to be further mined.

**Figure 7:**
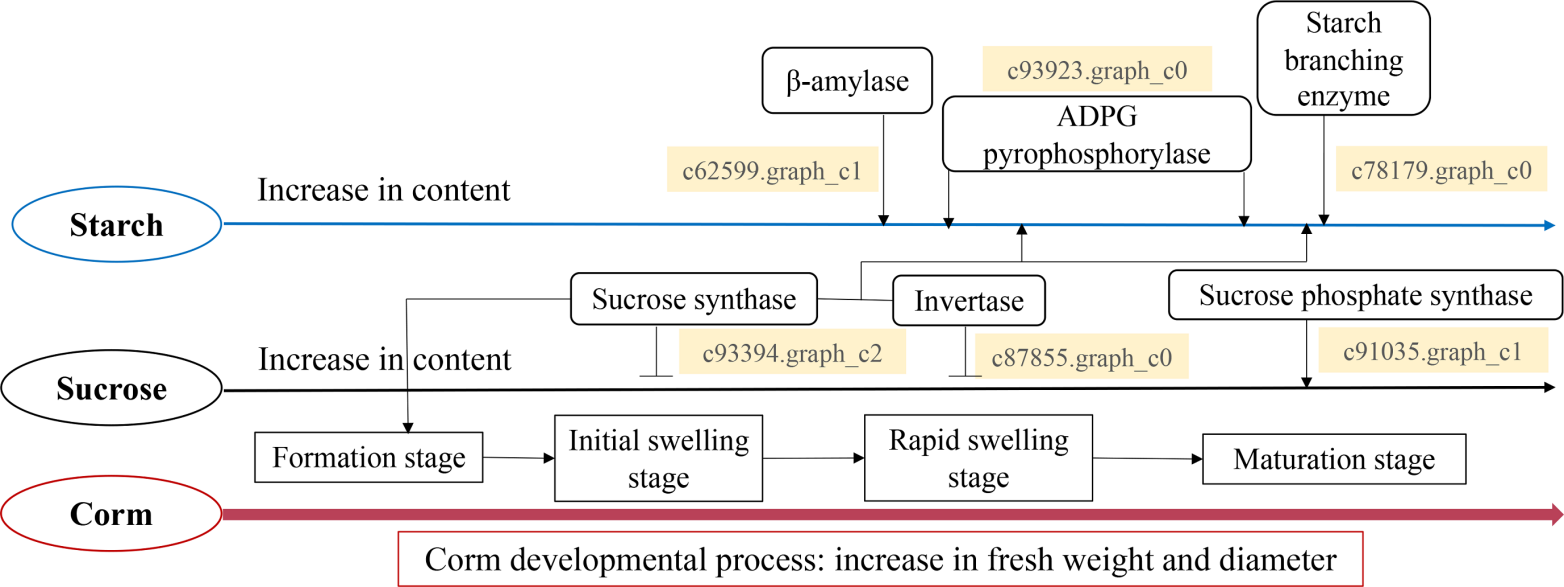
Proposed model for the starch and sucrose metabolism during corm development in freesia.

Taken all together, we propose a model for the starch and sucrose metabolism during corm development in freesia (Fig. 7). Corm development was paralleled with concomitant weight and size increase, together with the accumulation of starch, soluble sugar and sucrose. Though the dynamic pattern of the metabolizing enzymes differed, it was in general positively connected to the mode of starch and sucrose accumulation. Gene expression analysis of critical enzymes indicated that sucrose metabolism was regulated by SuSy, INV and SPS genes. The substantial expression of SuSy genes at the initial stage of corm formation boosted sucrose accumulation, while SPS played roles mainly during the mid- and late stages of corm development. Additionally, from corm swelling till maturation, INV and SuSy coordinately catalyzed sucrose breakdown to provide substrates for starch synthesis, resulting in starch accumulation. Starch metabolism was a comprehensive effect of *β*-AMY, SBE and APGase. Especially during the mid- and late developmental stages, gene expression of these enzymes rose remarkably, leading to rapid starch accumulation as energy reserve in mature corms. The proposed model is generally in line with some of the reported results for *Gladiolus hybridus* (Yuan et al., 2008), tulip (Miao et al., 2016b) and lily (Li et al., 2014).

## Conclusions

Herein, we investigated the dynamics of the major form of carbohydrates and related enzyme activities, and also profiled the transcriptome of freesia corms at four developmental stages. Obviously, corm development was paralleled with dynamics of starch and sucrose which were the comprehensive effects of carbohydrate-metabolizing enzymes. On this basis, corms at four representative sampling events were selected for transcriptome profiling based on RNA-seq. Transcriptomic results also showed that carbohydrate metabolism was the most active biological pathway with DEGs significantly enriched during corm swelling. Subsequently, DEGs from the transcriptome data encoding carbohydrate-metabolizing enzymes were correlated to the enzyme activities and carbohydrate accumulations and some critical genes involved in starch-sucrose metabolism were determined. The transcriptomic analysis thus provides further insights into molecular mechanisms underlying the metabolic process. To our knowledge, this is the first transcriptome analysis of starch and sucrose metabolism during corm formation and development in freesia. The results will shed light on elucidation of molecular mechanisms in corm formation and development and benefit for future improvements in breeding of freesia.

##  Supplemental Information

10.7717/peerj.11078/supp-1 Figure S1 GO Function classification of differentially expressed genes

10.7717/peerj.11078/supp-2 Figure S2 COG function classification of differentially expressed genes

10.7717/peerj.11078/supp-3 Figure S3 KEGG pathway enrichment analysis of differentially expressed genes (DEGs)

10.7717/peerj.11078/supp-4 Figure S4 Real-time PCR analysis of the six selected genes of key enzymes involved in starch and sucrose metabolism

10.7717/peerj.11078/supp-5 Figure S5 Expression pattern of 6 genes by RNA-seq and qRT-PCR

10.7717/peerj.11078/supp-6 Table S1 Corm developmental process and the morphology of *Freesia hybrida*‘SN Huangjin’

10.7717/peerj.11078/supp-7 Table S2 Sample sequencing statistics

10.7717/peerj.11078/supp-8 Table S3 Assembly result statistics

10.7717/peerj.11078/supp-9 Table S4 Statistics of unigene annotation

10.7717/peerj.11078/supp-10 Table S4 Putative genes corresponding to key enzymes in sucrose and starch metabolism

10.7717/peerj.11078/supp-11 Table S6 Primer sequences used for qRT-PCR

10.7717/peerj.11078/supp-12 Supplemental Information 1 Raw unigene data for transcriptome profiling

10.7717/peerj.11078/supp-13 Supplemental Information 2 Raw data for Figures 1, 2, and 6 and rt-PCR

## Additional Information and Declarations

### Data Availability

The following information was supplied regarding data availability: Raw sequence data are available at NCBI: SRR12576824, SRR12576823, SRR12576822, SRR12576821, SRR12576820, SRR12576819, SRR12576818, SRR12576817, SRR12576816, SRR12576815, SRR12576814, SRR12576813. Unigenes are also available at NCBI: GIYA00000000. Raw measurements for Figs. 1, 2, 6 and qRT-PCR are available in the Supplemental Files.
