# Supplementary material for: Enzymatic and transcriptomic analysis reveals the essential role of carbohydrate metabolism in freesia (Freesia hybrida) corm formation
Source: PeerJ. 2021 Mar 19;9:e11078. doi: 10.7717/peerj.11078 (PMC7983857; doi:10.7717/peerj.11078)
Supplement: Figure S3 [file peerj-09-11078-s003.pdf]

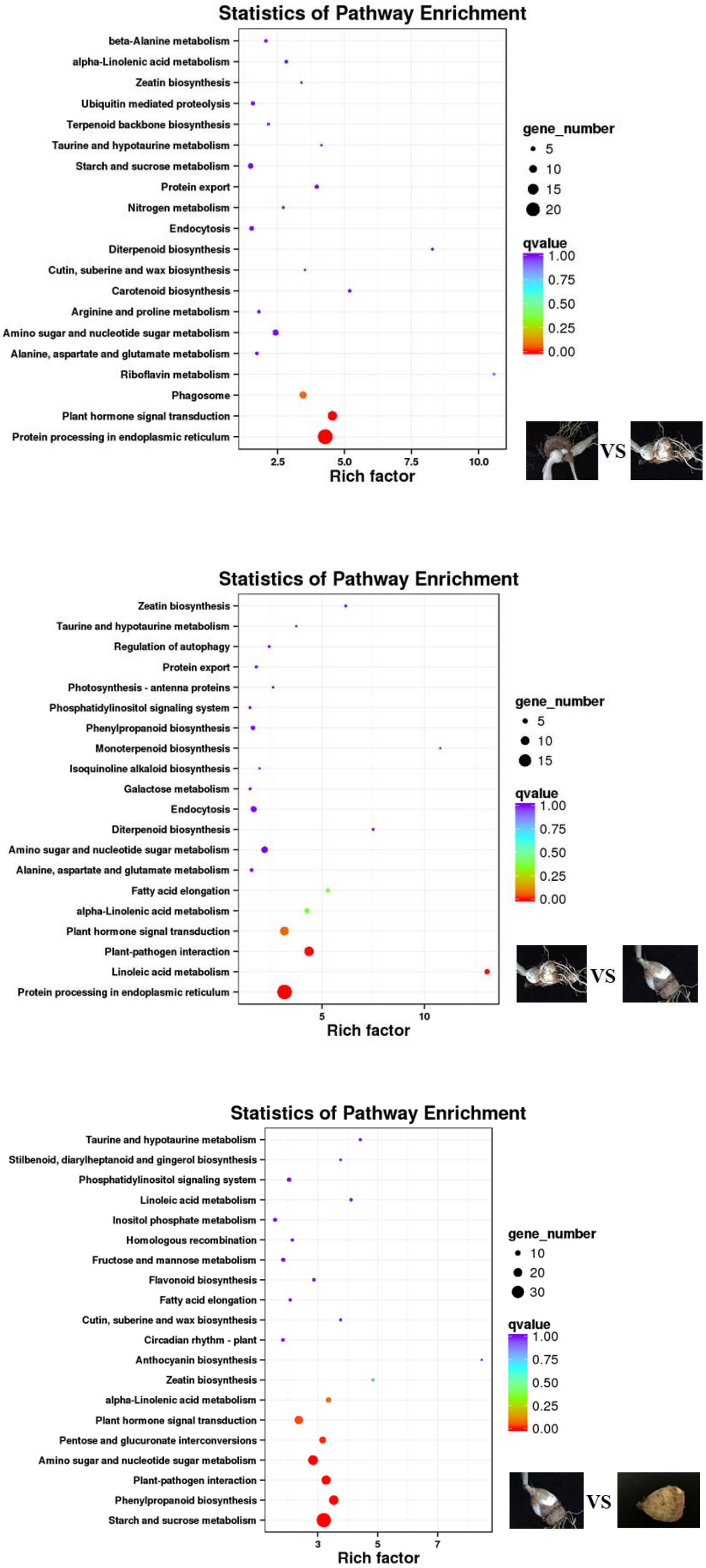

Fig.S3 KEGG pathway enrichment analysis of differentially expressed genes (DEGs). Top: 60 d vs. 90 d; middle: 90 d vs. 120 d; bottom: 120 d vs. 190 d; Rich factor: the ratio of the number of DEGs to all unigenes enriched in each pathway
