# Supplementary material for: Enzymatic and transcriptomic analysis reveals the essential role of carbohydrate metabolism in freesia (Freesia hybrida) corm formation
Source: PeerJ. 2021 Mar 19;9:e11078. doi: 10.7717/peerj.11078 (PMC7983857; doi:10.7717/peerj.11078)
Supplement: Figure S4 [file peerj-09-11078-s004.pdf]

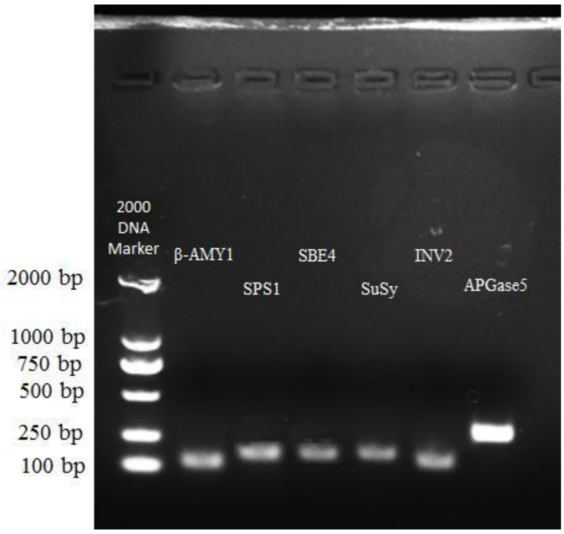

Fig. S4 Primer specificity and amplicon size of 6 selected primer pairs

β-AMY: β-amylase; SPS: sucrose phosphate synthase; SEB: starch branching enzyme; SuSy: sucrose synthase; INV: invertase; AGPase: adenosine diphosphoglucose pyrophosphorylase
