# Supplementary material for: Enzymatic and transcriptomic analysis reveals the essential role of carbohydrate metabolism in freesia (Freesia hybrida) corm formation
Source: PeerJ. 2021 Mar 19;9:e11078. doi: 10.7717/peerj.11078 (PMC7983857; doi:10.7717/peerj.11078)
Supplement: Figure S5 [file peerj-09-11078-s005.pdf]

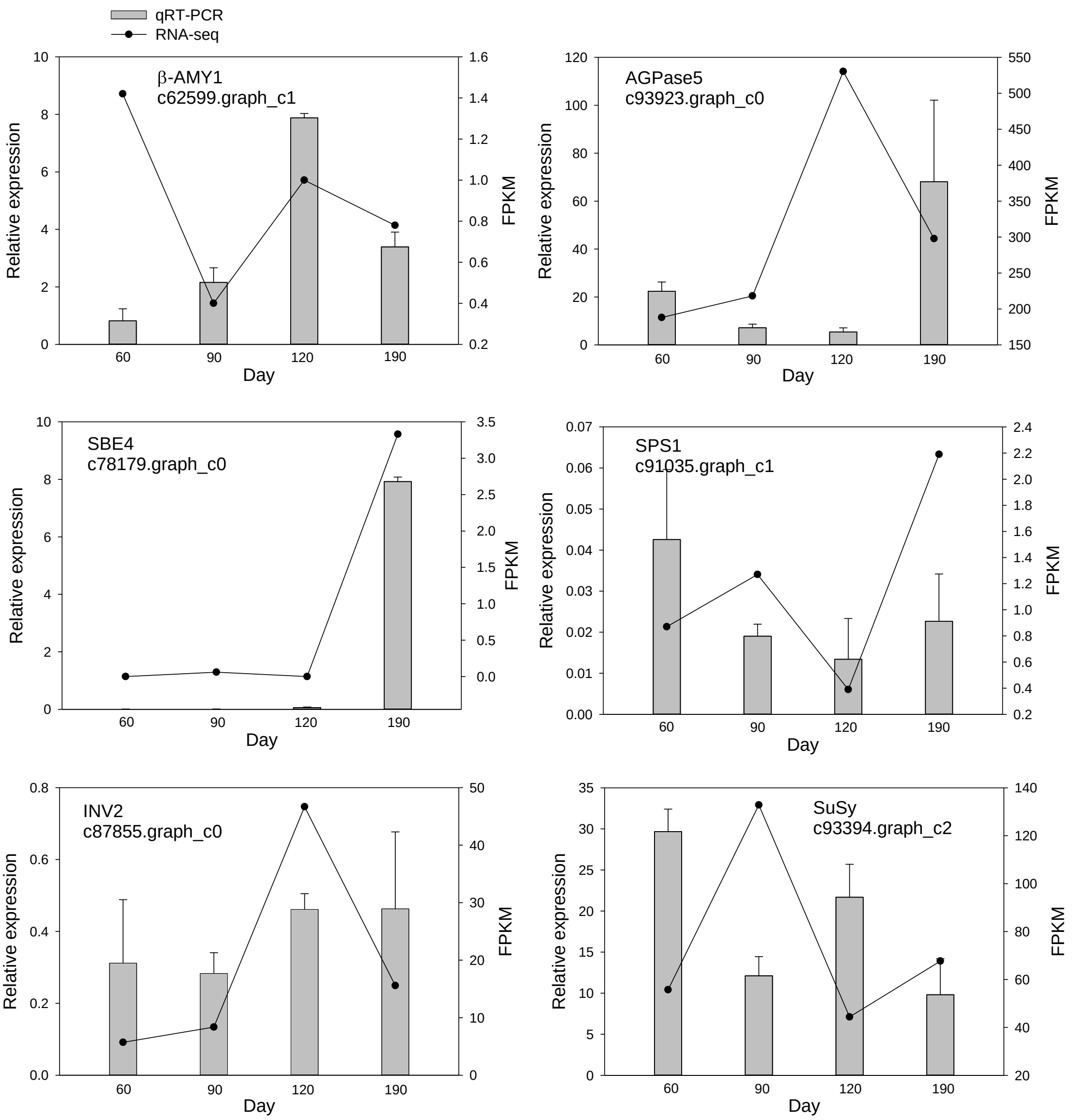

Fig. S5 Expression pattern of 6 genes by RNA-seq and qRT-PCR. FPKM: fragments per kilobase of transcript per million mapped reads; AMY: amylase; AGPase: adenosine diphosphoglucose pyrophosphorylase; SEB: starch branching enzyme; SPS: sucrose phosphate synthase; INV: invertase; SuSy: sucrose synthase
