# Supplementary material for: Enzymatic and transcriptomic analysis reveals the essential role of carbohydrate metabolism in freesia (Freesia hybrida) corm formation
Source: PeerJ. 2021 Mar 19;9:e11078. doi: 10.7717/peerj.11078 (PMC7983857; doi:10.7717/peerj.11078)
Supplement: Table S1 [file peerj-09-11078-s006.docx]

Tab.S1 Corm developmental process and the morphology of *Freesia hybrida* ‘SN Huangjin’

| Day after planting | 60～90 d | 90~120 d | 120~140 d | 140~190 d |
| --- | --- | --- | --- | --- |
| Plant developmental stage | Five-leaf to eight-leaf stage | Eight-leaf to flower bub stage | Flower bub to blossoming stage | Late blossoming to withering stage |
| New corm developmental stage | Formation stage | Initial swelling stage | Fast swelling stage | Maturation stage |
| Plant morphology | 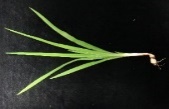 | 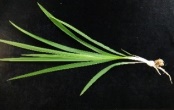 | 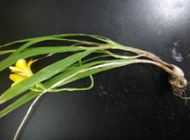 | 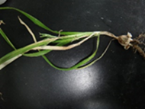 |
| Corm morphology | 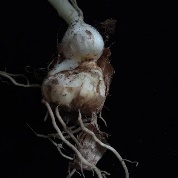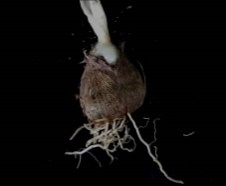 | 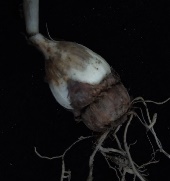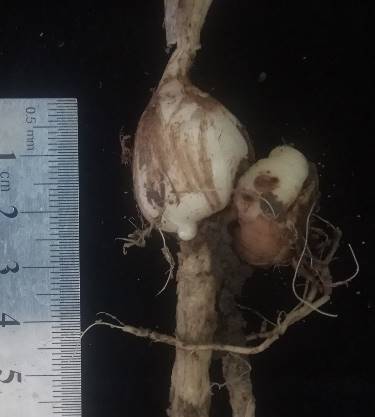 | | 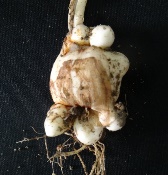 |
