# Supplementary material for: Enzymatic and transcriptomic analysis reveals the essential role of carbohydrate metabolism in freesia (Freesia hybrida) corm formation
Source: PeerJ. 2021 Mar 19;9:e11078. doi: 10.7717/peerj.11078 (PMC7983857; doi:10.7717/peerj.11078)
Supplement: Table S2 [file peerj-09-11078-s007.docx]

Tab. S2. Sample sequencing statistics

| **Sampling time** | Sample No. | Read Number | Base Number | GC Content (%) | %≥Q30 |
| --- | --- | --- | --- | --- | --- |
| 60 d | Replicate 1 | 24,059,895 | 7,186,077,668 | 48.62 | 90.41 |
|  | Replicate 2 | 25,067,234 | 7,503,785,210 | 48.75 | 90.28 |
|  | Replicate 3 | 22,944,214 | 6,870,994,810 | 48.01 | 90.21 |
| 90 d | Replicate 1 | 20,591,342 | 6,161,624,114 | 48.01 | 90.84 |
|  | Replicate 2 | 21,219,968 | 6,345,930,834 | 48.53 | 90.95 |
|  | Replicate 3 | 22,081,563 | 6,602,994,876 | 48.39 | 91.14 |
| 120 d | Replicate 1 | 22,274,056 | 6,650,519,592 | 48.86 | 91.23 |
|  | Replicate 2 | 20,626,622 | 6,171,667,094 | 47.94 | 91.42 |
|  | Replicate 3 | 23,265,121 | 6,966,566,312 | 48.51 | 90.54 |
| 190 d | Replicate 1 | 24,074,854 | 7,210,221,252 | 48.08 | 89.73 |
|  | Replicate 2 | 23,731,802 | 7,107,068,318 | 48.34 | 89.99 |
|  | Replicate 3 | 25,910,476 | 7,757,734,348 | 47.57 | 89.87 |
