# Supplementary material for: Enzymatic and transcriptomic analysis reveals the essential role of carbohydrate metabolism in freesia (Freesia hybrida) corm formation
Source: PeerJ. 2021 Mar 19;9:e11078. doi: 10.7717/peerj.11078 (PMC7983857; doi:10.7717/peerj.11078)
Supplement: Table S3 [file peerj-09-11078-s008.docx]

Tab. S3. Assembly result statistics

| **Length Range (nt)** | **Transcript** | **Unigene** |
| --- | --- | --- |
| 200-300 | 42,741(22.95%) | 38,361(37.98%) |
| 300-500 | 34,235(18.38%) | 24,846(24.60%) |
| 500-1000 | 37,129(19.93%) | 16,316(16.15%) |
| 1000-2000 | 41,502(22.28%) | 11,660(11.54%) |
| 2000+ | 30,652(16.46%) | 9,816(9.72%) |
| Total Number | 186,259 | 100,999 |
| Total Length | 204,842,160 | 77,966,713 |
| N50 Length | 1,858 | 1,507 |
| Mean Length | 1099.77 | 771.96 |
