# Supplementary material for: Enzymatic and transcriptomic analysis reveals the essential role of carbohydrate metabolism in freesia (Freesia hybrida) corm formation
Source: PeerJ. 2021 Mar 19;9:e11078. doi: 10.7717/peerj.11078 (PMC7983857; doi:10.7717/peerj.11078)
Supplement: Table S4 [file peerj-09-11078-s009.docx]

Tab. S4. Statistics of unigene annotation

| **#Anno_Database** | **Annotated_Number** | **300<=length<1000** | **length>=1000** |
| --- | --- | --- | --- |
| COG_Annotation | 13753 | 3867 | 5719 |
| GO_Annotation | 25142 | 8089 | 8733 |
| KEGG_Annotation | 15743 | 5127 | 6092 |
| KOG_Annotation | 23015 | 7243 | 9079 |
| Pfam_Annotation | 28454 | 8034 | 13988 |
| Swissprot_Annotation | 24441 | 7765 | 11095 |
| eggNOG_Annotation | 39635 | 12883 | 14414 |
| Nr_Annotation | 41175 | 13900 | 14943 |
| All_Annotated | 44405 | 14919 | 15062 |
