# Supplementary material for: Enzymatic and transcriptomic analysis reveals the essential role of carbohydrate metabolism in freesia (Freesia hybrida) corm formation
Source: PeerJ. 2021 Mar 19;9:e11078. doi: 10.7717/peerj.11078 (PMC7983857; doi:10.7717/peerj.11078)
Supplement: Table S4 [file peerj-09-11078-s010.docx]

Tab. S5. Putative genes corresponding to key enzymes in sucrose and starch metabolism

| Enzymes | Number of genes | Homologue ID | Gene ID | FPKM value | | | | |
| --- | --- | --- | --- | --- | --- | --- | --- | --- |
|  |  |  |  | Formation stage | Initial swelling stage | Rapid swelling stage | | Maturation stage |
| β-Amylase | 2 | β-AMY1  β-AMY2 | ***c62599.graph_c1***  c62599.graph_c0 | 1.42  0 | 0.40  0 | | 1  0.22 | 0.78  0.62 |
| Invertase | 6 | INV1  INV2  INV3  INV4  INV5  INV6 | c97994.graph_c3  ***c87855.graph_c0***  c103826.graph_c0  c101131.graph_c0  c44649.graph_c0  c61341.graph_c0 | 14.59  5.71  1.10  4.14  0.44  0.57 | 14.22  8.36  0.87  5.30  0  0 | | 59.39  46.68  2.37  17.36  0  0 | 26.95  15.58  4.31  5.81  0  0.28 |
| Sucrose phosphate synthase | 10 | SPS1  SPS2  SPS3  SPS4  SPS5  SPS6  SPS7  SPS8  SPS9  SPS10 | ***c91035.graph_c1***  c84397.graph_c1  c163996.graph_c0  c101027.graph_c0  c78039.graph_c0  c91035.graph_c0  c84397.graph_c2  c103258.graph_c0  c66639.graph_c0  c102171.graph_c0 | 0.87  3.29  0  40.74  3.23  0.36  4.83  42.52  0  0.82 | 1.27  4.80  0.30  99.57  3.21  0.92  4.51  92.64  0.34  6.31 | | 0.39  5.45  0  41.63  3.24  0.28  6.19  76.53  0  3.47 | 2.19  8.75  0.26  144.2  5.76  1.67  4.73  184.2  0  14.88 |
| Sucrose synthase | 1 | SuSy | ***c93394.graph_c2*** | 55.73 | 132.8 | | 44.37 | 67.68 |
| Starch branching enzyme | 10 | SBE1  SBE2  SBE3  SBE4  SBE5  SBE6  SBE7  SBE8  SBE9  SBE10 | c94559.graph_c0  c164483.graph_c0  c130587.graph_c0  ***c78179.graph_c0***  c141987.graph_c0  c101693.graph_c0  c168688.graph_c0  c101577.graph_c0  c123622.graph_c0  c177432.graph_c0 | 35.17  0  1.06  0  0  410.59  0  3.39  0  0.26 | 33.36  0.88  0  0.06  0  361.5  0.84  9.65  0  0 | | 32.34  0  0  0  0.14  238.7  0  6.81  0.4  0 | 38.89  0  0  3.33  0  90.31  0  7.6  0  0 |
| ADPG pyrophosphorylase | 8 | APGase1  APGase2  APGase3  APGase4  APGase5  APGase6  APGase7  APGase8 | c163904.graph_c0  c87667.graph_c1  c173485.graph_c0  c74641.graph_c0  ***c93923.graph_c0***  c91512.graph_c0  c75933.graph_c0  c74834.graph_c0 | 0.47  1.43  0.37  1.79  188.08  791.12  1.19  3.64 | 0  2.17  0  0.2  218.1  538.4  0.09  1.51 | | 0  2.69  0  0.13  530.46  1351  0.07  2.16 | 0  2.27  0  0.12  297.8  536.2  0.62  2.19 |

Note: Genes in bold and italic were selected for qRT-PCR.
