# Supplementary material for: Enzymatic and transcriptomic analysis reveals the essential role of carbohydrate metabolism in freesia (Freesia hybrida) corm formation
Source: PeerJ. 2021 Mar 19;9:e11078. doi: 10.7717/peerj.11078 (PMC7983857; doi:10.7717/peerj.11078)
Supplement: Table S6 [file peerj-09-11078-s011.docx]

Tab. S6. Primer sequences used for qRT-PCR

| Homologue | Gene | Primer | Length (bp) |
| --- | --- | --- | --- |
| β-AMY1 | c62599.graph_c1 | ATGATCCCCCACCAAACAT | 121 |
|  |  | CATTGGGAGTGGTCAATGTAG |  |
| INV2 | c87855.graph_c0 | CCTCGACAGGTCGTAGTC | 126 |
|  |  | CGGCGATTCTTCTCC |  |
| SPS1 | c91035.graph_c1 | AGATCCGGATTATGCTACCC | 186 |
|  |  | GGTGTTGCCTTGCTCTTATC |  |
| SuSy | c93394.graph_c2 | CGTCCTAGACCTGGAGTC | 173 |
|  |  | CGAGGGAGGGACTCAT |  |
| SBE4 | c78179.graph_c0 | CCAAGGGGAGTCCAA | 142 |
|  |  | GCATCGCTCGGTCAA |  |
| APGase5 | c93923.graph_c0 | GCTCAGCAGAGTCCAGAG | 293 |
|  |  | AGCTGGTCCCCCTTAG |  |

AMY: amylase; INV: invertase; SPS: sucrose phosphate synthase; SuSy: sucrose synthase; SEB: starch branching enzyme; AGPase: adenosine diphosphoglucose pyrophosphorylase
