# Supplementary material for: Enzymatic and transcriptomic analysis reveals the essential role of carbohydrate metabolism in freesia (Freesia hybrida) corm formation
Source: PeerJ. 2021 Mar 19;9:e11078. doi: 10.7717/peerj.11078 (PMC7983857; doi:10.7717/peerj.11078)
Supplement: Figure S2 [file peerj-09-11078-s002.pdf]

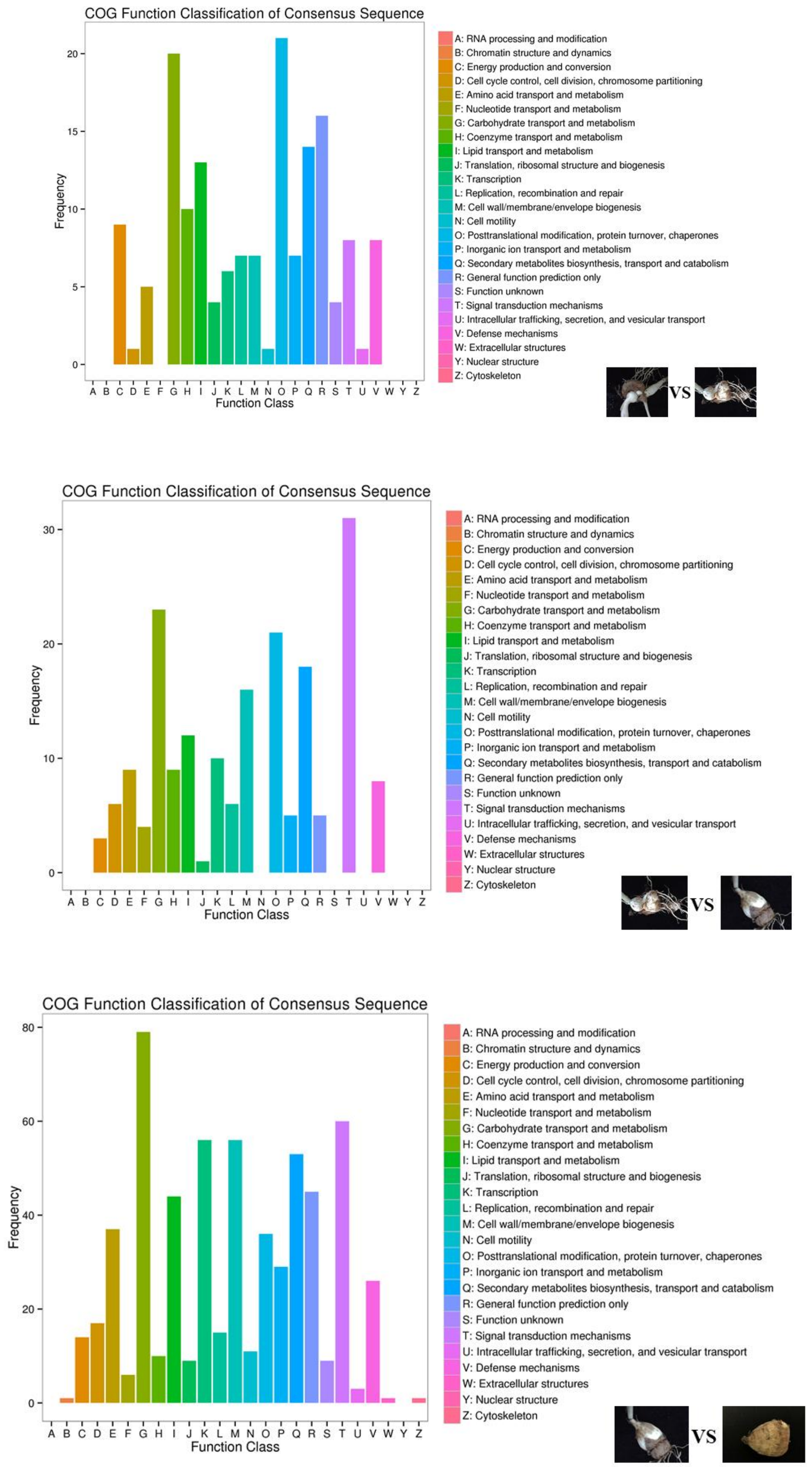

Fig. S2 COG function classification of differentially expressed genes. above: 60 d vs. 90 d; middle: 90 d vs. 120 d; below: 120 d vs. 190 d
